# Phytochemical Delivery Through Transferosome (Phytosome): An Advanced Transdermal Drug Delivery for Complementary Medicines

**DOI:** 10.3389/fphar.2022.850862

**Published:** 2022-02-23

**Authors:** Rong-Ping Chen, Vivek P. Chavda, Aayushi B. Patel, Zhe-Sheng Chen

**Affiliations:** ^1^ Department of Endocrinology, Zhujiang Hospital, Southern Medical University, Guangzhou, China; ^2^ Department of Pharmaceutics and Pharmaceutical Technology, L M College of Pharmacy, Ahmedabad, India; ^3^ Pharmacy Section, L.M. College of Pharmacy, Ahmedabad, India; ^4^ Department of Pharmaceutical Sciences, College of Pharmacy and Health Sciences, St. John’s University, Queens, United States

**Keywords:** transferosome, phytosome, complementary medicine (CAM), phytochemicals, transdermal drug delivery

## Abstract

Transdermal drug delivery aims to create a safe and effective method of administering drugs through the skin that attracts a lot of attention and investment due to the constant progress in the field. Transferosomes are flexible or malleable vesicles (having almost the same structure as liposomes but with better skin penetration properties) discovered initially in the early 90s. The name transferosomes, which means “carrying bodies,” is coined from the Latin phrase “Transferee,” which means “to carry through,” and the Greek term “soma,” meaning “body.” In comparison to typical herbal extracts, phytosomes (Transferosomes) are created by attaching specific herbal extracts to phosphatidylcholine, resulting in a formulation with increased solubility and, hence, better absorption, resulting in improved pharmacokinetic and pharmacodynamic features of the entrapped drugs. We are using the word phytosomes and transferosomes interchangeably as we have consolidated vesicular delivery of herbal drugs through skin. In this mini-review, we have demonstrated the enormous potential of developing nanotechnology to deliver bioactive phytochemicals, with a special emphasis on phytosomes (Transferosomes) as a unique lipid-based nanocarrier for transdermal drug delivery.

## Introduction

Transdermal drug delivery system (TDDS) is an appealing option to oral drug administration and is poised to give an alternative to hypodermic injection as well. People have been applying herbal extracts and chemicals to their skin for thousands of years for therapeutic purposes, and in the contemporary period, a wide range of topical formulations have been produced to treat local indications (Alkilani et al., 2015). In comparison to the oral route, transdermal administration provides several benefits. It is utilized particularly when the liver has a substantial first-pass impact that might cause medications to be metabolized early. Transdermal administration also offers advantages over hypodermic injections, which are uncomfortable, create hazardous medical waste, and risk of disease transmission through needle re-use, particularly in impoverished nations (Jeong et al., 2021).

**FIGURE 1:**
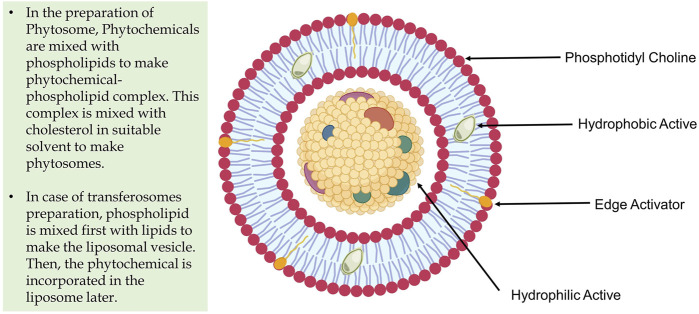
Structural feature of Transferosome and Phytosome.

The introduction of transferosome (phytosome) nanotechnology can transform the existing state of external therapeutic phytochemicals delivery. The fundamental obstacle in translating phytochemicals’ therapeutic potential to a clinical context is their extremely low absorption rate and limited penetration through biological barriers (Alharbi et al., 2021). Phytosomes, as lipid-based vesicular nanocarriers, perform an essential role in improving the pharmacokinetic and pharmacodynamic characteristics of herbal-derived polyphenolic chemicals, making this nanotechnology a prospective tool for the creation of novel topical formulations. Because of their distinct physicochemical properties, phytochemicals may be able to penetrate biological barriers more easily with the use of this nanosized vesicular drug delivery method, and ultimately enhancing their bioavailability (More et al., 2021). Medicinal plants and their phytochemicals are currently widely used as a treatment for a variety of ailments. Nonetheless, their low bioavailability and selectivity may restrict their therapeutic use. As a result, bioavailability is regarded as a significant problem in improving bio-efficacy in transporting dietary phytochemicals (Barani et al., 2021). However, with enhanced membrane flexibility, ultra-deformability, and soft nature, the basic structure of a transferosome is ideal approach for transdermal phytochemical delivery for local and systemic drug action (Pawar et al., 2016). However, phospholipid interacts with the phytochemicals via the configuration of an H-bond in between the polar head of the phospholipid and the polar functions of the phytochemical constituents. Flexibility is achieved by incorporating an edge activator (surfactants) into the cholesterol-phospholipid bilayers (Figure 1). Transferosomes are especially effective for transporting medicines with limited solubility through the epidermis. The ability of phytoconstituents derived from herbal sources to penetrate the epidermis or any other semi-permeable barrier is relatively restricted (Chavda et al., 2021; Opatha et al., 2020).

## Phytochemical Delivery Through Transferosome (Phytosome)

Transferosomes are applied topically in a non-occluded manner and have been proven to permeate into the stratum corneum (lipid lamellar areas), leading to the skin’s hydration (Benson, 2006). An osmotic gradient develops due to the evaporation of skin water by the body heat, which acts as a driving force to transfer the drug from the site of application to the targeted area, either locally or for systemic action (Sudhakar et al., 2021; Naik et al., 2006). Furthermore, transferosomes enhance the functions of the skin by improving enzyme balance, hydration, and collagen structure (Opatha et al., 2020). Because of their hydrophilicity, polyphenols have lower absorption and lipid solubility, restricting their *in vivo* action. Several flavonoid molecules attach firmly to the phospholipid components of phytosomes. Transferosomes are infused with phytoconstituents with limited permeability and incorporated into dosage forms for topical applications such as the transdermal patches to facilitate better bioavailability with site-specific action, which is referred to as ‘Phytosomes’. In short, phytosomes are transferosomes with an infused phytoconstituent or phytochemical (Romero and Morilla, 2013). The difference between transferosome and phytosome can also be understood by their method of preparation though the composition is identical (Figure 1). Phytosomes continue to outperform herbal extracts in improving its pharmacokinetics and pharmacodynamics (Lu et al., 2019). The transferosomes–herbal complex shows greater affinity to the skin phospholipid component, increasing the topical formulation’s lipid solubility (Alharbi et al., 2021).

**FIGURE 2:**
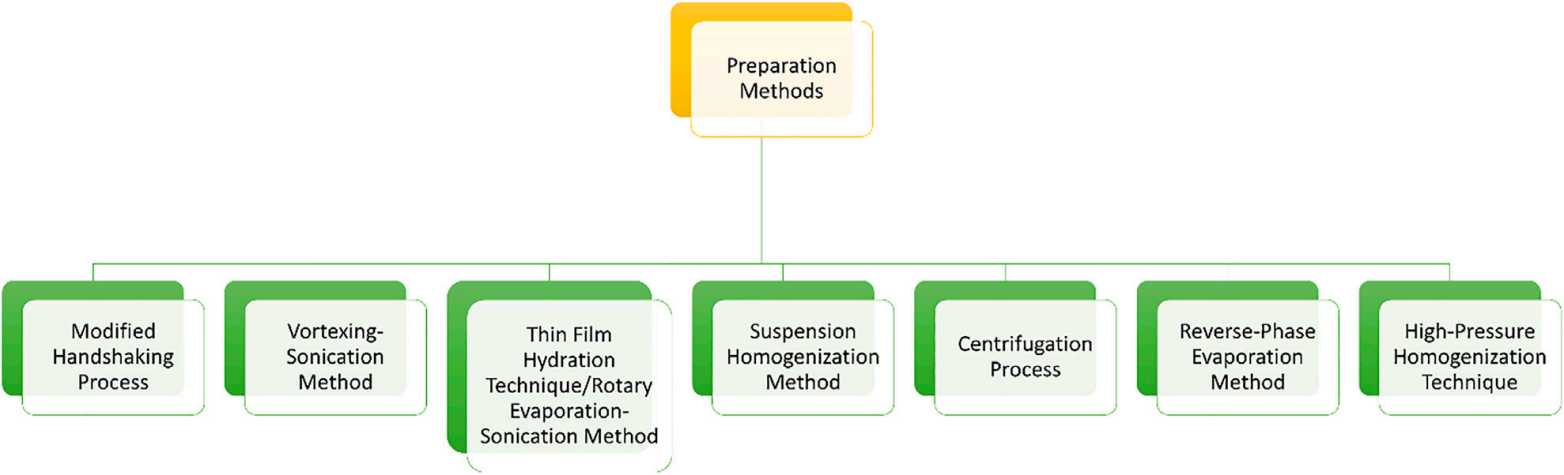
Method of preparation for Transferosomes (phytosomes).

Transferosomes majorly involve the ingredients like amphipathic ingredients (combination of hydrophilic and lipophilic molecules like soy phosphatidylcholine), surface activators (e.g. surfactants), alcohol, and water (Jiang et al., 2018). The preparation method was determined based on the composition of phytosomes, such as drug-carrying capacity and suitable transporter with optimal stability and deformability (Kumar, 2018). Numerous techniques for creating phytosomes have been presented, and classified such as the film hydration method, (also known as a rotational evaporation sedimentation method) is a frequently utilized preparation process for transferosomes and phytosomes, and it enables more topical penetration through the skin as compared to other modified approaches (Figure 2) (Singh et al., 2017). Solvent evaporation is popular and widely utilized approach for generating phospholipid vesicles. In addition to the traditional procedure, methods such as freeze-thaw, centrifugation, reverse-phase evaporation, high-pressure homogenization, and suspension homogenization are used to engineer the phytochemical loaded transferosomes (Opatha et al., 2020). Transfersome characterization variables (Table 1) such as vesicle shape and size, size distribution, polydispersity index, zeta potential, number of vesicles per cubic mm, entrapment efficiency, degree of deformability, and skin permeability measurements are useful for optimising the transfersomal formulation (Opatha et al., 2020).

**TABLE 1:** Charecterization methods for transferosome.

Evaluation parameter	Determination method	References
Number of vesicles/cubic mm	(The total number of phytosomes/transfersomes counted × dilution factor)/The total number of squares counted	Chauhan and Tyagi (2018)
Vesicle size distribution	Photon correlation spectroscopy or Dynamic light scattering method	Chauhan and Tyagi, (2018)
Zeta potential	Electrophoretic mobility technique	Chauhan and Tyagi (2018)
Degree of deformability	D = J (rv/rp) Where rv and rp indicate the vesicle size and the barrier pore size respectively, while J remains for the amount of suspension squeezed out in 5 min	Chauhan and Tyagi (2018)
Entrapment efficiency (EE)	% EE = The amount of drug entrapped ×100/The total amount of drug added	Ascenso et al. (2015)
*In vitro* skin permeation studies	Human skin is ideal for permeation studies but due to less availability, various animal models and animal skin were examined for permeation study, including porcine, rat, mouse Guinea pig, primate, and snake skins. Systemic membranes such as Start M^®^ and Franz cell model are also widely used for determination	Singh et al. (2017)
*In vitro* drug release	Extrusion method or Franz diffusion cells are used	GM et al. (2008)
Stability studies	The vesicle size and structure changes concerning time is taken into consideration, for that transmission electron microscope (TEM) or dynamic light scattering methods (DLS) are employed	Ascenso et al. (2015)

Transfersomes have the potential to open up a whole new world of possibilities for efficient drug administration. The following are the benefits of using transfersomes as vesicle-based TDDS,

• High vesicle deformability allows medications to be transported through the skin without significant loss of intact vesicles and can be utilized for both topical and systemic therapies (Moawad et al., 2017).
• Transfersomes are a natural alternative for producing continuous drug release as well as predictable and prolonged activity duration of the drug (Opatha et al., 2020).
• Transfersome carriers are made up of hydrophilic and hydrophobic moieties, resulting in a one-of-a-kind drug carrier system capable of delivering therapeutic drugs with a wide range of solubility (Bnyan et al., 2018).
• Avoiding first-pass metabolism, which is a key disadvantage of oral medication delivery, results in improved drug bioavailability (Moawad et al., 2017).
• Because they are composed of natural phospholipids and edge activators, they appear to be biocompatible and biodegradable (Li et al., 2015).
• They can improve bioactive agents' site selectivity and increase transdermal flow.
• Reduce the drug’s unwanted side effects while also protecting it from metabolic destruction (Bnyan et al., 2018).
• They have the benefit of being created from pharmaceutically approved substances and using established processes, but they must be developed and optimized on an individual basis (Opatha et al., 2020).
• It is straightforward to scale up because of a rapid and simple manufacturing technique (Li et al., 2015).

Transfersomes offer several advantages, but they also have some restrictions,

• The high cost of raw materials and costly equipments for manufacturing impacts the costing of the final drug product. As a result, because it is very inexpensive, phosphatidylcholine is the most often utilized lipid component (Iskandarsyah et al., 2018).
• Another barrier for using transfersomes as a drug delivery mechanism is the difficulty in obtaining pure natural phospholipids. As a result, synthetic phospholipids might be employed as a substitute (Grit and Crommelin, 1993; Iskandarsyah et al., 2018).
• Because of their propensity for oxidative destruction, transfersomes are thought to be chemically unstable. Transfersome oxidation can be considerably reduced by storing a product at a low temperature and keeping it away from light. Transfersome storage performance can be improved with post-preparation processing such as freeze-drying and spray-drying (Grit and Crommelin, 1993).

Currently, many clinical trials are in progress for the transdermal delivery of various drugs using transferosomes as a carrier however, all are on synthetic analogues (Opatha et al., 2020). Various phytochemicals are known to be delivered with the help of transferosomes as a carrier. Avadhani et al., in 2017, formulated transferosomes of epigallocatechin-3-gallate and hyaluronic using a modified thin-film hydration method, followed by a high-pressure homogenization technique. This work resulted in the enhancement of their efficacy as antioxidants (Avadhani et al., 2017). In 2019, Wu et al. studied the antioxidant drug resveratrol (RSV) and its resveratrol loaded transferosomes, including its antioxidant assays, *in vitro* transdermal delivery analysis, and cell viability assay (Wu et al., 2019). Formulation containing RSV transferosomes showed enhancement in stability, bioavailability, and safety of resveratrol (Szulc-Musioł and Sarecka-Hujar, 2021). Research conducted by Jiang et al. states that the topical delivery of anticancer agents like paclitaxel in melanoma chemotherapy can be done by paclitaxel transferosomes embedded within oligopeptide hydrogels (Jiang et al., 2018). Vincristine transferosomes have shown an increase in their permeation capability through the skin and improvement in their lymph targeting ability (Lu et al., 2007; Pahwa et al., 2021). Transferosomes are found to improve the stability and the efficacy of anti-inflammatory drugs. Transferosomes embedded with curcumin were developed and have shown an increase in their permeation capability and bioavailability (Anita et al., 2021). A study on the antiarthritic activity of capsaicin-loaded transferosomes in arthritic rats showed better inhibitory activity and the desired therapeutic concentration of the drug at the target site (Jain et al., 2021). Similarly, Ines Castangia and collegues have prepared the transferosome encapsulating Jabuticaba (*Myrciaria jaboticaba*) extract having flavonoids having better wound-healing in human keratinocytes (Castangia et al., 2021). Caffeine transferosomes also showed greater permeation across the stratum corneum of the skin and an increase in permeation of hydrophilic caffeine through hair follicles (Abd et al., 2016). Various other phytochemicals like embelin and colchicine can also be delivered transdermally by forming its transferosomes. Numerous experts have discovered novel techniques and generated phytosome compositions (Table 2).

**TABLE 2:** Patents on phytosomes for the phytochemical delivery.

Name of patent	Development	Patent No
Phospholipid complexes derived from olive fruits or leaves with enhanced bioavailability	The bioavailability of olive fruit/leaf extracts is improved when it is employed in phospholipid complexes	EP1844785
Curcumin phospholipid complex with improved bioavailability	Curcumin phospholipid complexes have a larger systemic level of primary agent than simple curcumin	WO 2009/101551
Wound healing and skin treatment, with thymosin β-4	Preparation is comprising thymosin β4 for wound repair	US/2007/0015698
Oral formulations for cellulite therapy	*Centella asiatica* triterpenes, *Vitis vinifera* extracts, and *Ginkgo biloba* flavonoids in free or complexed form with phospholipids are used in topical pharmaceutical preparation	US7691422
For the management of asthmatic and allergic disorders, formulations including *Ginkgo biloba* metabolites	Constituents of *Ginkgo biloba* fractions for the therapy of asthma and allergic diseases	EP1813280

## Conclusion and Future Prospects

As new phytochemicals are discovered, studies on their medicinal advantages in a biological setting will be continually updated. Future research could look into combining phytosomes with several other phytochemicals or combining drugs and phytochemicals in the same nano-vesicle to produce stimulatory activity. Phytosomes are identical to liposomes and have equivalent skin permeability and stability profiles. In phytosomes, however, the phospholipid interfaces with the phytochemicals through the generation of an H-bond between the polar head of the phospholipid and the polar capabilities of the bioactive molecules. In compared to liposomes, this significantly improves the stability and skin penetration of phytochemicals. Clinical trials are still inadequate to judge the bioactivities of specific compositions, but the conclusive data for these compositions is positive, and experts are encouraged to continue their studies in this sector. Clinical trials on standardized products that demonstrate improved efficacy relative to non-formulated components or extracts will be critical in driving interest in these advancements. All the research works carried out in the recent past for the phyochemical loaded lipidic vesicles i.e., transferosome depicts the potential of this formulation approach to tackle the challenges associated with phyochemicals for their successful transdermal delivery for local and systemic action. Further, clinical research data of such drug delivery platform in the near future will unveils the future potential use of such drug delivery platform.
